# Predictive Maintenance of Norwegian Road Network Using Deep Learning Models

**DOI:** 10.3390/s23062935

**Published:** 2023-03-08

**Authors:** Muhammad Umair Hassan, Ole-Martin Hagen Steinnes, Eirik Gribbestad Gustafsson, Sivert Løken, Ibrahim A. Hameed

**Affiliations:** Department of ICT and Natural Sciences, Norwegian University of Science and Technology (NTNU), 6009 Ålesund, Norway; omsteinn@stud.ntnu.no (O.-M.H.S.); eirikgg@stud.ntnu.no (E.G.G.); lsivert@stud.ntnu.no (S.L.)

**Keywords:** predictive maintenance, anomaly detection, deep learning, highway predictive maintenance

## Abstract

Industry 4.0 has revolutionized the use of physical and digital systems while playing a vital role in the digitalization of maintenance plans for physical assets in an optimal way. Road network conditions and timely maintenance plans are essential in the predictive maintenance (PdM) of a road. We developed a PdM-based approach that uses pre-trained deep learning models to recognize and detect the road crack types effectively and efficiently. We, in this work, explore the use of deep neural networks to classify roads based on the amount of deterioration. This is done by training the network to identify various types of cracks, corrugation, upheaval, potholes, and other types of road damage. Based on the amount and severity of the damage, we can determine the degradation percentage and have a PdM framework where we can identify the intensity of damage occurrence and, thus, prioritize the maintenance decisions. The inspection authorities and stakeholders can make maintenance decisions for certain types of damages using our deep learning-based road predictive maintenance framework. We evaluated our approach using precision, recall, F1-score, intersection-over-union, structural similarity index, and mean average precision measures, and found that our proposed framework achieved significant performance.

## 1. Introduction

According to Statistics Norway, there are a total of 54,899 km of county and national roadways in Norway that the Norwegian Public Road Administration (NPRA) is responsible for monitoring and ensuring are well-maintained. Predictive maintenance (PdM) techniques are designed to determine the conditions of roadways in order to estimate when maintenance should be performed [1]. Compared to traditional maintenance activities, such as time-based preventative maintenance, PdM is conditions-based, where maintenance is carried out based on real-time estimations of the degradation state of the roadway infrastructure fostering safety and cost-savings [2]. PdM is a proactive maintenance approach that aims to predict equipment or asset failures before they occur and take preventative actions to minimize the downtime and costs associated with repairs [3]. This approach is widely used in many industries, including transportation and infrastructure.

In the context of roads, predictive maintenance can involve using various technologies to monitor and assess road conditions in real time, detect potential defects or damage, and predict when repairs or maintenance may be needed [4]. Some of the technologies that may be used for this purpose include sensors and internet-of-things (IoT) devices that can be embedded in the road surface to monitor factors, such as temperature, humidity, traffic volume, and pavement strain [5]. In Norway, road surface inspection vehicles are deployed to map the road surfaces two times a year to reduce risks associated with poor road quality and schedule maintenance. Inspection vehicles are equipped with camera systems to measure the road surface conditions. Each collected image is labeled with geolocation. Only a limited number of the collected images are manually analyzed based on reports from road users because the manual inspection is a very tedious, time-consuming, and inefficient process [1].

**Figure 1:**
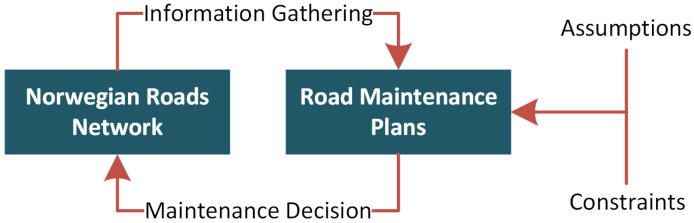
Conceptual design of PdM planning for road infrastructure.

The fourth industrial revolution (Industry 4.0) strongly emphasizes the integration of physical and digital systems [6,7]. PdM-based road inspection is a novel and demanding concept to maintain the road’s infrastructure. A thematic diagram of PdM for roads is available in Figure 1. The information from the road network is required for the maintenance plans and to make maintenance decisions. The assumptions define the maintenance plans, while constraints restrict the scope of the plans. Road networks re vital in enabling ground transport; consequently, the roads are directly linked to a wide range of economic and social activities. Therefore, proposing maintenance plans for roads is crucial to preserve both the capacity and value of road assets.

Machine learning (ML) and deep learning (DL) models have encouraged PdM-based approaches regarding maintenance decisions in road infrastructure [8]. This paper proposes using pre-trained DL models to classify and categorize road surface distress. Here, a set of DL models was trained to predict road conditions using a labeled dataset regarding the type and severity of the road crack. When deployed, the trained model can automatically analyze road conditions and decide which road sections are due for maintenance. In addition, it can be used to document road conditions for contracting and estimating expected costs accurately. Various variants of deep convolutional neural networks (CNNs) are used in our proposed work. A CNN uses convolution layers to extract features from an image. The result from a convolution layer is a feature map. A complete CNN is built up with several convolutional layers, one or more hidden layers, and an output layer. The fully-connected layers and output layers are tasked with making the final decision, i.e., road conditions in terms of distress types and severity.

For training and testing, NPRA provided two datasets for two different roads; 2483 and 626 images, respectively. Domain experts visually inspected the two datasets to identify and quantify existing road damage, distress types, and extent. Annotated images were then used for training, testing, and validating the proposed DL models. Four distress types were used in this work: alligator cracking, vertical cracking, horizontal cracking, and potholes. Due to the scarcity of labeled images, pre-trained transfer learning models, such as the faster R-CNN model [9] on the MS-COCO dataset [10] achieved the best accuracy. Precision and recall could be significantly improved if more labeled datasets are available. We used cloud computing services and APIs from Microsoft Azure. The developed API allows multiple images to be uploaded and renders damages in a heatmap for easy use.

The objective of this research is to make DL-based models accessible through a server, and the models should detect several types of road cracks in an effective way. We leveraged the pre-trained DL models for detecting the different road crack types. The pre-trained models performed better relative to other types of AI approaches for road crack detection. Pre-trained models were trained on large datasets [11] with diverse road conditions, which enabled them to recognize various patterns and features related to road cracks. This pre-training on large datasets helped to generalize the model’s ability to detect cracks in different road conditions and environments. Pre-trained models are often based on DL architectures, such as CNNs, which are specifically designed for image processing tasks [12]. This allows the model to extract and learn complex features from road images that can be difficult for traditional ML approaches to detect. These models are often fine-tuned on smaller datasets of road images with annotated cracks [13]. Pre-trained models are often available as open-source code and pre-trained weights, making it easier for researchers and developers to use and customize them for their specific needs [14]. This ease of access can save time and resources, allowing developers to focus on customizing the model for specific use cases. Overall, it has been demonstrated that pre-trained models perform better for road crack detection due to their ability to recognize complex patterns; moreover, they are highly flexible and can be easily customized.

The paper is organized as follows. The related works are presented in Section 2. We provide the details of data collection and methods used in this work in Section 3, while the framework development is discussed in Section 4. The evaluation metrics are presented in Section 5. The results are demonstrated in Section 6. Section 7 provides a brief discussion of our work. Finally, the concluding remarks are available in Section 8.

## 2. Related Works

The PdM plays a vital role in making early decisions to save maintenance costs. Li et al. [15] proposed a preventive maintenance-based intelligent decision-making model for asphalt roads using a particle swarm optimization and enhanced gated recurrent neural network. The use of image processing and ML techniques has opened up new channels for road crack detection methods. For example, Chun et al. [16] combined the image processing and naïve Bayes classifier for the categorization of road pavement cracks automatically. Zalama et al. [17] proposed a Gabor filter and AdaBoost training-based method to identify road damage, while another work [18] focused on integrating the traditional image processing and classification techniques for pavement crack detection. Some metaheuristic ML approaches are combined with image-processing algorithms to recognize road cracks automatically. In this regard, Hoang et al. [19] used the least-square support vector machine with an artificial bee colony algorithm to achieve the maximized accuracy in classifying different types of road cracks.

In addition to ML-based road crack detection approaches, DL-based models have also been deployed to classify road damage detection. Wu et al. [20] proposed a classification method using the U-Net architecture [21]. Qu et al. [22] developed a road crack detection method by using the fine-tuned LeNet-5 to classify original images and VGG-16 [23] to extract the features of road cracks in the detection phase. Li et al. [24] applied the adaptive-cost sensitive loss functions for imbalance dataset problems in road crack detection. They also developed a database of road pavement cracks in Beijing’s night scenes.

Djenouri et al. [25] proposed a method to detect road cracks using a graph convolutional neural network (GCNN). They computed the visual features of roads using scale invariant feature transformation (SIFT) and then analyzed a correlation between SIFT features of similar images. A genetic algorithm supervises their developed GCNN to optimize the hyperparameters of the network. They tested their model on seven different datasets. Fan et al. [26] also studied the road crack detection problem and proposed a residual attention-based UNet by introducing the balance loss. They solved the data imbalance problem of images in the road cracks datasets.

A mobile CNN-based approach for detecting cracks in the road’s surface is proposed by Dogan et al. [27]. The authors introduced a lightweight network based on MobileNetV2 that can be used in mobile devices to detect road cracks. They trained their model on Crack500 dataset [28]. In another work, Xu et al. [29] compared faster R-CNN and mask R-CNN for road crack detection. This work is closely related to our work because we also compare deep learning models for detecting road cracks. An encode–decoder-based transformer architecture is proposed to model the crack features in long-range dependencies [30]. A local enhancement module was added to the transformers to add the capacity to learn from local features. The authors manually annotated the dataset to improve the robustness of their proposed LETNet architecture.

Fang et al. [31] proposed an attention-based TransUNet for crack detection in road surfaces. The TransUNet takes the detailed texture information of detected cracks from the shallow layers and passes it to deep layers through skip connections. The authors added a transformer block in the second last convolution stage to explicitly model the long-range dependency of the image regions. They evaluated their method on four road crack datasets. Sun et al. [32] detected the road cracks under noise conditions. They produced a dataset with multiple noise crack images called NCD. After that, they leveraged an adaptive bilateral filtering algorithm to reduce the noise influence. Ultimately, they designed a network with two new modules forming a feature pyramid structure with a feature enhancement strategy.

A distribution equalization learning methodology for road crack detection is presented in [33]. The authors proposed a truncated expansion-based methodology for data augmentation to relieve sample imbalance and developed weighted cross-entropy loss to avoid ill-posed classifier issues. They proposed auxiliary interaction loss to alleviate the detected image region cracks. The authors in [34] proposed a multi-scale classification network for road crack detection by focusing on the feature maps of CNN. They added the weighted values of pixels in corresponding image regions with different scales to learn features for road cracks.

A method by Guo et al. [35] detected the road cracks by considering the image edges as additional features and adapting the image gradient to produce precise crack boundaries. The authors introduced an edge adaptation module in their proposed method and used 3D convolution for handling feature map relations in different channels. Dung et al. [36] proposed a fully convolutional network (FCN)-based methodology for the semantic segmentation of road cracks. Similar to our work, they used the pre-trained network for image classification on the public road dataset. Then they trained the FCN network for the semantic segmentation of cracks on a subset of annotated images.

## 3. Data and Methods

**Figure 2:**
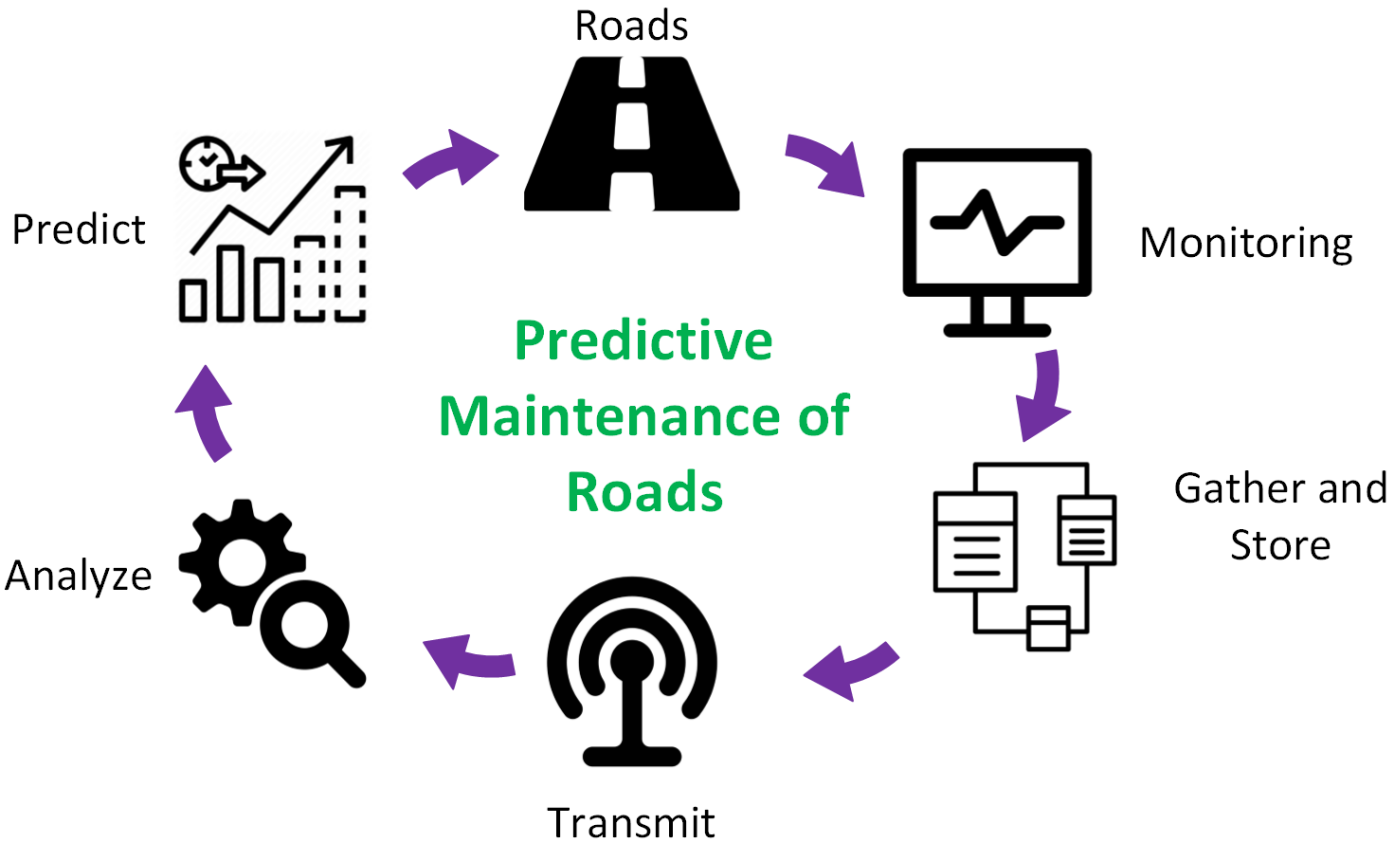
PdM-based framework for the maintenance of road networks.

Recent technological advancements have made it feasible to conveniently monitor the road’s infrastructure using different cameras, recognizing the road conditions by detecting specific damage types to execute maintenance plans and decisions. A PdM-based framework for monitoring and detecting road cracks is presented in Figure 2.

The collected road data were monitored and saved in a database; they were then sent for analysis. We used different ML- and DL-based pre-trained models to predict road conditions, and maintenance plans were made for areas where the damage was detected.

The NPRA inspects Norwegian roads (urban and county roads). During these inspections, NPRA gathers data by taking pictures of the roads and scanning them with a laser scanner. The data used in this work were gathered during these inspections. During the first part of the PdM application, the images from these inspections were used to train and verify a CNN to identify cracks in the tarmac surface. The data were collected in two phases: First, the roads were scanned with a LiDAR scanner that could scan with a high density of points to analyze the depth of cracks and other road damages. Second, the gathered images from two cameras mounted in front of the car (containing both the tarmac surface and the surroundings) were used to analyze the environment close to roads. The road administration also experimented with images behind the car, focusing only on the road surface at a top-down angle. Both datasets were manually inspected to extract images containing road damages before annotation. The images were then divided into training and evaluation sets. The details of the datasets are as follows.

**Veidekke dataset.** These images were taken as part of a prototype setup where the NPRA is implemented on all vehicles. The cameras are mounted at the vehicle’s rear, angled approximately 20 degrees to the road surface, providing a top-down view. Due to the images being part of a prototype setup, only 2144 images were gathered. Each image was RGB with a resolution of 2046×2046. The size of each image was roughly 500 kb and contained metadata with GPS coordinates. We split this dataset into 561 training and 65 evaluation images.

**P18 dataset.** The P18 images were taken during the annual road inspection. Therefore, the road administration databases contained several million images accumulated over several years. Two cameras mounted at the front of the car gathered the images. Each image was RGB with a resolution of 2703×1018. The size of each image was roughly 350 kb and contained metadata with GPS coordinates. We split the P18 dataset into 1932 training and 551 evaluation images.

There were several meta-architectures used for road crack detection. We used the single shot detector (SSD) [37] and faster R-CNN [9] in our PdM-based road crack detection framework.

**Figure 3:**
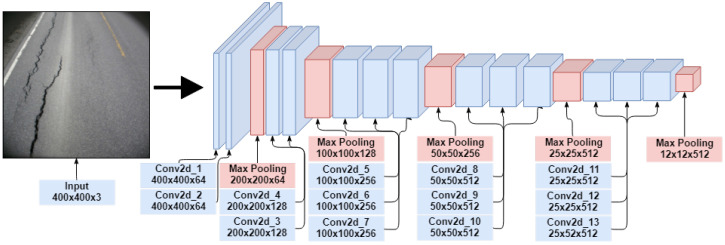
An illustration of the convolution and max-pooling layers of the VGG-16 network used in the CNN.

**SSD.** A single shot detector is a feed-forward convolutional network, which means that classification and detection are done in a single forward pass in the network (built to be simple relative to other approaches). It was proposed to be faster than Faster R-CNN while being more accurate [37]. SSD has become the standard in object detection, though its impressive speed will (to some extent) limit accuracy [38]. The base network of an SSD architecture is VGG-16 without the fully connected layers, which leaves 15 convolution layers (see Figure 3). Training the SSD network requires an input image with labeled ground truth boxes.

**Faster R-CNN.** It consists of two modules; a deep fully convolutional network for the region proposal and the detector from the fast R-CNN model. The region proposal network (RPN) takes an image as input and gives a set of rectangular object proposals with the corresponding objective scores as output. These outputs are then fed through the detector network, determining the class and score. The model can be trained in four steps using an alternative training method, such as (i) training the RPN, (ii) training the faster R-CNN detector network using the region proposals from the RPN, (iii) using the trained detector to initialize a new RPN training session, where the shared convolutional layers become fixed while tuning only the layers unique to the RPN, and (iv) keeping the convolutional layers fixed, fine-tuning the unique layers of the detector network. The computational cost is drastically reduced by sharing the convolutional layers between the RPN and the detector network, resulting in reduced processing times.

## 4. Framework Development

Within TensorFlow’s object detection API, we created a generalized method of configuring pre-trained models called the pipeline configuration. The pipelines consist of a protocol buffer (*protobuf*) file that holds the settings for both the training and evaluation. The *protobuf* file dictates which meta-architecture and feature extractor the model consists of. Further, it decides both parameters and metrics in training and evaluation. It also defines the input paths of the training, evaluation, and label map data. Additionally, the pipeline configuration determines if the model should start training from a pre-trained checkpoint or train from scratch.

The deployment of trained models with TensorFlow Serving for a website was achieved using Docker, which utilizes the Google Remote Procedure Call (gRPC) protocol to communicate with a client script that provides images for prediction.

**Figure 4:**
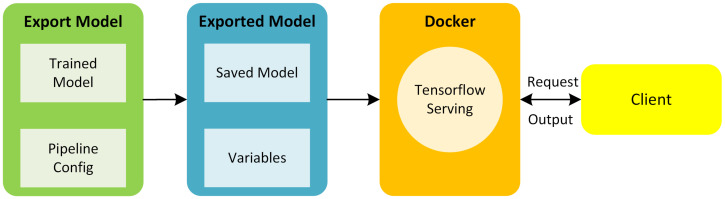
Process flow for PdM deployment.

As seen in Figure 4, a trained model and its configuration file were exported into a saved model and a variables directory. Preferably, the trained model should be frozen, as this eliminates the meta-data and variables that are not necessary. Encapsulating the model in a single file is not as computationally intensive. However, using the NVIDIA docker makes it possible to utilize the GPU on the virtual machine. The serving image was built within Docker. Moreover, the exported model was committed to Docker. Finally, a TensorFlow model server was started to manage the model service. By running the model server, the model was accessible from the client’s scripts.

**Serving clients.** Several clients were created to use the deployed object detection models. A back-end script in the web page that received uploaded images from the user before sending them to the served model and receiving predictions was the most prominent. Additionally, a script to auto-label road cracks was created. Both scripts were connected through gRPC to Docker. The web page was intended to receive images from users, predict road damage, and mark damage intensity on a map. A back-end script on the web page had to request predictions from the served model.

## 5. Experimental Evaluation

We used the following metrics to evaluate the performances of deep learning models for road crack detection.

**Precision.** Precision is the ratio between the correctly classified examples and the number of times the system has classified examples of a particular class.

(1) $$\;\mathrm{Precision}\;=\frac{\;\mathrm{True}\;\mathrm{Positive}\;}{\;\mathrm{True}\;\mathrm{Positive}\;+\;\mathrm{False}\;\mathrm{Positive}\;}$$

**Recall.** Recall is the ratio between correctly classified examples and the total number of examples for a class.

(2) $$\;\mathrm{Recall}\;=\frac{\;\mathrm{True}\;\mathrm{Positive}\;}{\;\mathrm{True}\;\mathrm{Positive}\;+\;\mathrm{False}\;\mathrm{Negative}\;}$$

**F1-score.** The F1-score is a combination of precision and recall and is calculated by taking the harmonic mean value from precision and recall. When calculating the mean between ratios, the harmonic mean is more intuitive than the arithmetic mean and is used when calculating the F1-score.

(3) $$\;F1\text{-}\mathrm{score}\;=\frac{2\times \;\mathrm{Precision}\;\times \;\mathrm{Recall}\;}{\;\mathrm{Precision}\;+\;\mathrm{Recall}\;}$$

**Intersection Over Union (IoU).** IoU is a metric used to calculate the similarity between two arbitrary shapes $A,B\subseteq S\in R^{n}$. The IoU score is calculated using the following formula:

(4) $$\mathrm{IoU}=\frac{\left|A\cap B\right|}{\left|A\cup B\right|}$$

**Mean average precision.** In object detection, IoU is the primary evaluation metric. It is used to measure the degree of overlap between the ground truth, the labeled test data, and the predicted bounding box. For object detection, the equation can be simplified as follows:

(5) $$\mathrm{IoU}=\frac{TP}{FP+TP+FN}$$

where

- TP—True Positive
- FP—False positive
- FN—False negative.

mAP is a mean average precision score that requires an IoU threshold of at least 0.5, following Equation 6, and is used to calculate mAP in our work.

(6) $$\mathrm{mAP}=\frac{1}{N}\sum_{i=1}^{N}{\mathrm{AP}}_{i}$$

**Structural similarity index.** Measuring quality differences in images is a difficult task. The perceived quality of the human visual system can be different than measured pixel value differences and the signal-to-noise ratio. SSIM offers a method to quantify image degradation as perceived changes in structural information. Given two non-negative image signals, the similarity measure can serve as a quantitative measurement of the quality of the second image [39]. The SSIM index is based on three comparisons: luminance, contrast, and structure. The luminance comparison is a function of the estimated mean intensity of the luminance difference between the two signals, denoted as l(x,y), where the mean intensity is given by

(7) $$\mu_{x}=\frac{1}{N}\sum_{i=1}^{N}x_{i}$$

Signal contrast is estimated by taking the standard deviation. In discrete form, this is given by

(8) $$\sigma_{x}={\left(\frac{1}{N-1}\sum_{i=1}^{N}{\left(x_{i}-\mu_{x}\right)}^{2}\right)}^{\frac{1}{2}}$$

The contrast comparison denoted by c(x,y) is the comparison of $\sigma_{x}$ and $\sigma_{y}$. The structure comparison denoted as s(x,y) is given by the signals normalized by their standard deviation.

(9) $$\frac{x-\mu_{x}}{\sigma_{x}}$$

and

(10) $$\frac{y-\mu_{y}}{\sigma_{y}}$$

The three measures were combined into an SSIM given in the following general form.

(11) $$SSIM\left(x,y\right)={\left[l\left(x,y\right)\right]}^{\alpha }\cdot {\left[c\left(x,y\right)\right]}^{\beta }\cdot {\left[s\left(x,y\right)\right]}^{\gamma }$$

where α,β,γ>0 are weights used to adjust the relative importance of the components. The index ranges from 0 to 1, where 0 indicates complete dissimilarity and 1 indicates perfectly identical patches [40]. If X and Y are the images to be compared, computed as matrices of pixels, y, and x are a subset of local square windows located at the same spatial position in both images [40]. SSIM is defined for local square windows of an image and can be computed to evaluate the global image similarity by taking the mean SSIM for the entire image, also known as MSSIM.

## 6. Results

### 6.1. Object Detection

#### 6.1.1. Model 1—Faster R-CNN Inception ResNet V2 Astrous COCO

Model 1 was pre-trained on the MS-COCO dataset [10]. Additionally, it was fine-tuned for 10,000 epochs on the Veidekke dataset provided in this work. In Table 1, it can be seen that Model 1 achieved precision and recall of 67% and 79%, respectively, occurring 1765 times in the training set and 164 in the evaluation set, providing a decent base for the model to learn. Table 2 presents the confusion matrix of Model 1.

**Figure 5:**
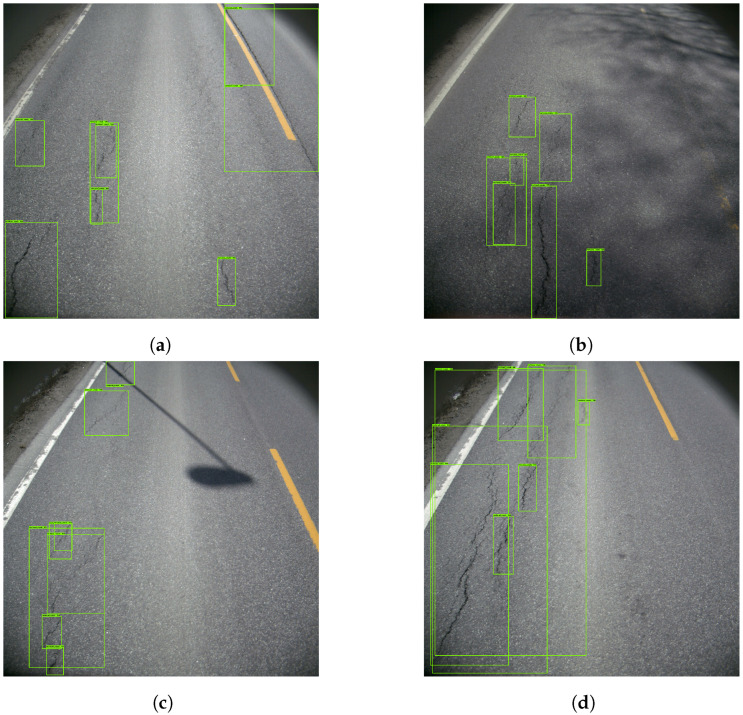
Examples of poor vertical crack detection. The green boxes represents the vertical cracks.

**Figure 6:**
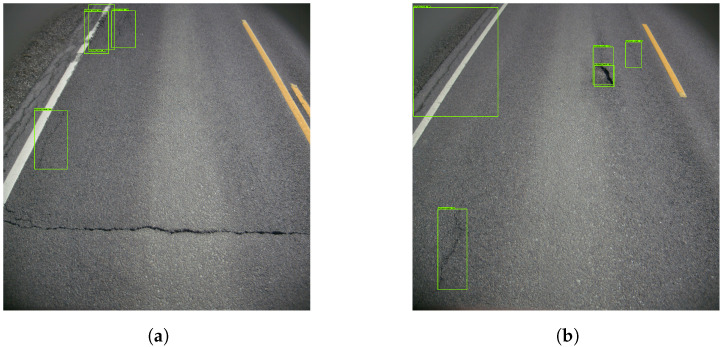
Examples of missed cracks. The green boxes represents the vertical cracks.

Figure 5 presents four examples, where the model correctly determined the presence of vertical cracks. It did, however, detect the same cracks several times. This resulted in a cluttered image filled with detection boxes. Given the low representation of other damages, the model struggled to detect them. Figure 6 provides insight into that specific problem. In Figure 6a, the horizontal crack is completely ignored. Figure 6b depicts a pothole that is mistakenly interpreted as a vertical crack.

**Figure 7:**
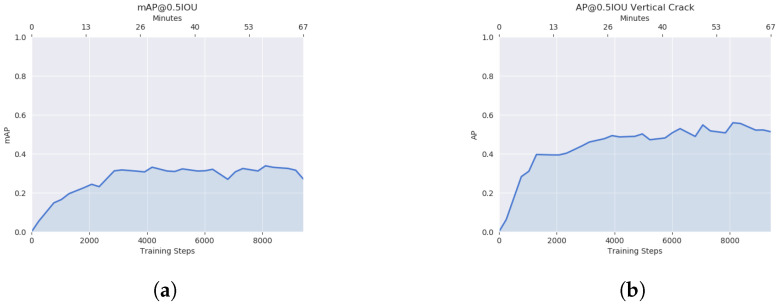
mAP and AP of the ‘vertical crack’ in Model 1. (**a**) Mean average precision. (**b**) Average precision of the ‘Vertical Crack’.

As can be seen in Figure 7a, the mAP convoluted at roughly 30%. Likewise, Figure 7b shows the average precision of ‘vertical crack’ detection to be slightly above 50%.

**Figure 8:**
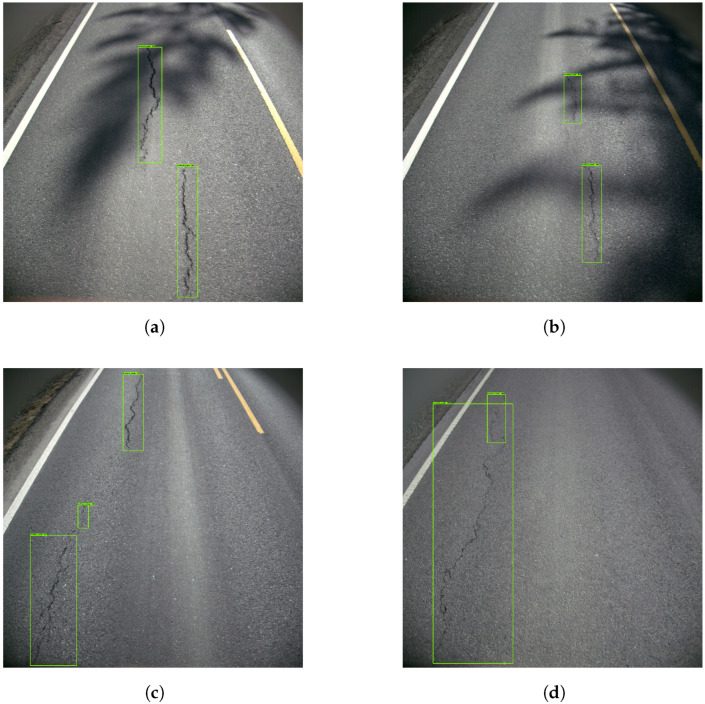
Examples of good vertical crack detection. The green boxes represents the vertical cracks.

The resulting images from Model 1 showed that the model recognized vertical cracks consistently. Figure 8 shows four examples of good vertical crack detection. In Figure 8a,b, the model correctly marks the vertical cracks despite shadows obscuring the cracks, making the task more challenging. Further, Figure 8c,d show the model detecting both larger and smaller cracks.

#### 6.1.2. Model 2—Faster R-CNN Inception ResNet V2 Astrous COCO

Model 2 was pre-trained on the MS-COCO dataset, and fine-tuned for 100,000 time steps on P18. Model 2 achieved an accuracy of 24%. It can also be seen that the models were able to reach 50% average accuracy for the ‘horizontal crack’. The model stopped improving after 30,000 timesteps.

Table 3 shows the respective precision and recall results produced by the evaluation calculated at a 50% confidence and the IoU threshold. Table 3 shows that while ‘vertical crack 1’ is the most represented class, it scores lower than the horizontal crack, which has a smaller sample size. The confusion matrix in Table 4 shows that the model predicted 334 instances of damage without there being any damage present. Similarly, 580 labeled damages were missed by the model. Further inspection revealed that ‘vertical crack 1’ was often mistaken for an ‘alligator crack’ or a ‘vertical crack 2’. This occurred 56 and 35 times. Moreover, 46 instances of ‘vertical crack 2’ damages were mistaken as ‘vertical crack 1’.

**Figure 9:**
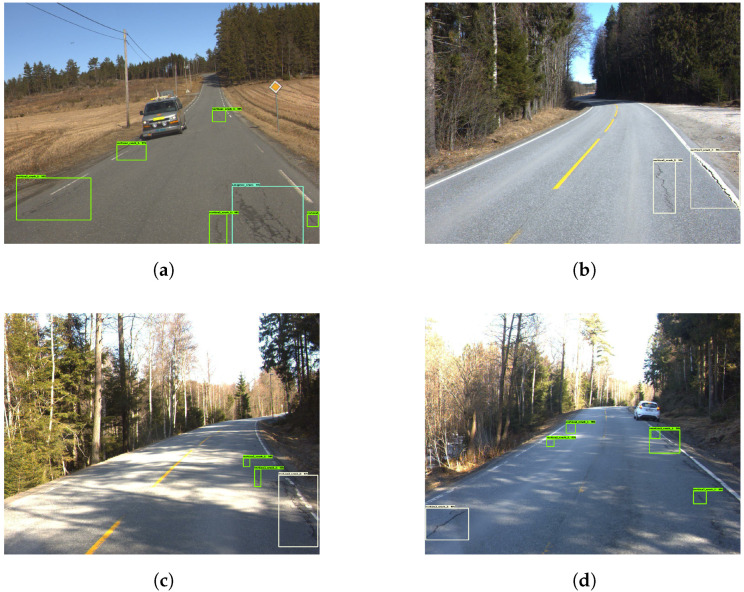
Good damage detection. The green and yellow boxes represent the vertical cracks while cyan colored boxes are the horizontal cracks.

Figure 9 shows examples of good damage detection with the model. Figure 9a shows that the model correctly detects five vertical cracks, as well as one ‘alligator crack’. Figure 9b shows the model detecting two ‘vertical crack 2’ instances. Moreover, both Figure 9c,d show the model detecting ‘vertical crack 1’ and ‘vertical crack 2’, despite shadows covering parts of the roads.

**Figure 10:**
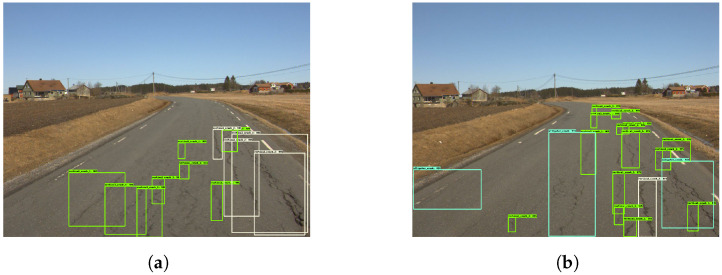
Cluttered detection—Model 2. The green and yellow boxes represent the vertical cracks while cyan colored boxes are the horizontal cracks.

The model struggles when an image contains several cracks that are in close proximity to each other, as seen in Figure 10. Through closer inspection of both Figure 10a,b, it becomes apparent that the reason for the intertwined boxes stems from the high frequency of cracks. The model subsequently misses several cracks in both figures. At first glance, the processed images look cluttered and incorrect. A closer inspection shows that the predicted boxes do in fact represent cracks.

**Figure 11:**
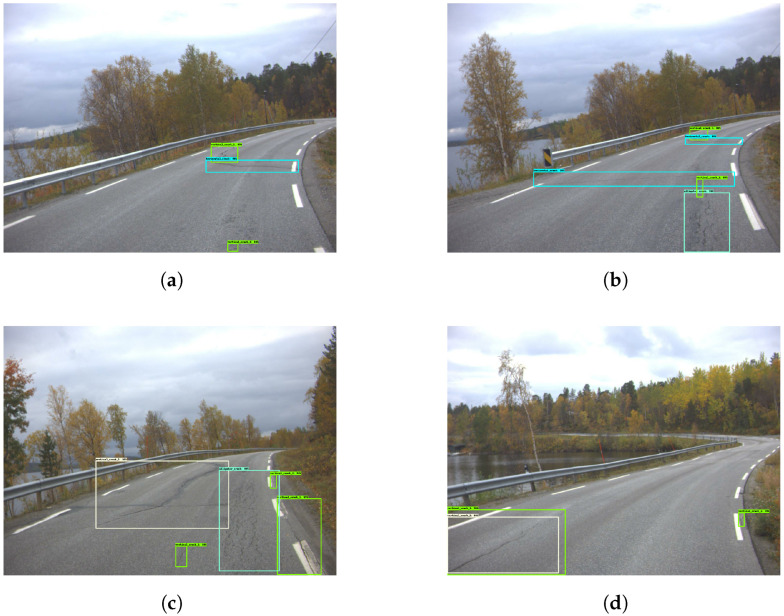
Damage detection—Model 2. The green and yellow boxes represent the vertical cracks while cyan colored boxes are the horizontal cracks.

As shown in Figure 11a,b, the model was good at distinguishing between different classes, despite the low accuracy scores and the mixed results of the confusion matrix. Moreover, Figure 11c shows how the model can miss distinct cracks, such as the horizontal crack depicted. At times, the model detects the same crack as two instances as seen in Figure 11d.

In total, 580 out of 1564 labeled damages were not detected by the model. Moreover, 334 predicted damages were non-existent.

#### 6.1.3. Model 3—Faster R-CNN ResNet 101 KITTI

Model 3 was pre-trained on the KITTI dataset [41] and fine-tuned on P18. The model achieved 15% mAP during evaluation. Table 5 shows Model 3 achieving relatively good precision and poor recall scores.

The confusion matrix located in Table 6 shows that 608 ‘vertical crack 1’ damages were missed. In 130 instances, the model wrongly predicted ‘vertical crack 1’ for non-existing damages. Further, the model mistook ‘vertical crack 1’ for ‘vertical crack 2’ 28 times. Similarly, 44 of the ‘vertical crack 2’ instances were classified as ‘vertical crack 1’. This means that roughly 25% of the more severe vertical cracks were predicted as less severe types of vertical damage. In total, 752 out of 1564 labeled damages were not detected by the model. Finally, a total of 165 predicted damages were non-existent.

**Figure 12:**
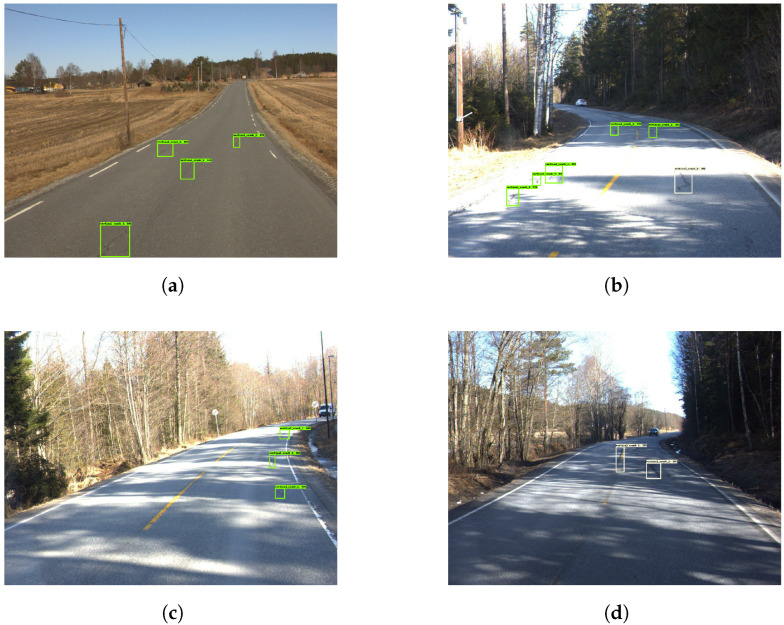
Good damage detection—Model 3. The green and yellow boxes represent the vertical cracks.

**Figure 13:**
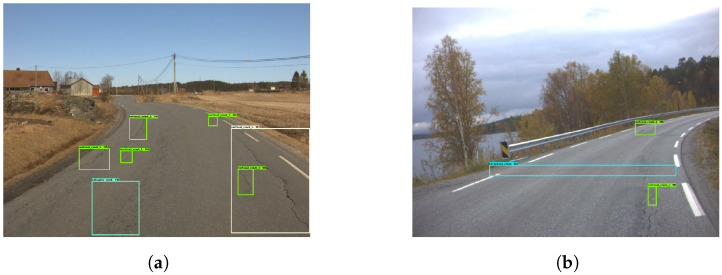
Low sensitivity in damage detection—Model 3. The green and yellow boxes represent the vertical cracks while cyan colored boxes are the horizontal cracks.

**Figure 14:**
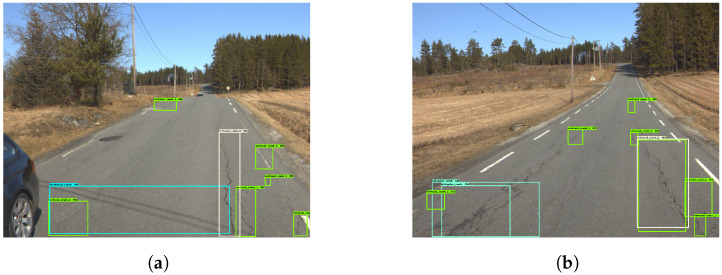
Poor damage detection—Model 3. The green and yellow boxes represent the vertical cracks while cyan colored boxes are the horizontal cracks.

When looking through the images presented in Figure 12, it appears that the model is fairly good at detecting cracks covered by shadows. Figure 13 shows that the model has low sensitivity, therefore resulting in a lot of smaller damages being missed. Figure 14 shows how the model missed several larger and more substantial cracks. It also mistakenly classified a shadow as a crack, as shown in Figure 14a.

#### 6.1.4. Model 4—Faster R-CNN Inception ResNet v2 Atrous Oidv4

Model 4 was pre-trained on the Oidv4 dataset [42], and fine-tuned on P18. It achieved a mAP of 14%. Through further evaluation, in Table 7, it is evident that while precision is high, recall is low. This results in a low F1-score, hence the poor performance. Further inspection of the confusion matrix, in Table 8, shows that 829 of 1564 labeled damages were not detected by the model. This means that only 46.9% of the labeled damages were recognized, not considering whether or not they were classified correctly. Finally, a total of 163 predicted damages were non-existent.

**Figure 15:**
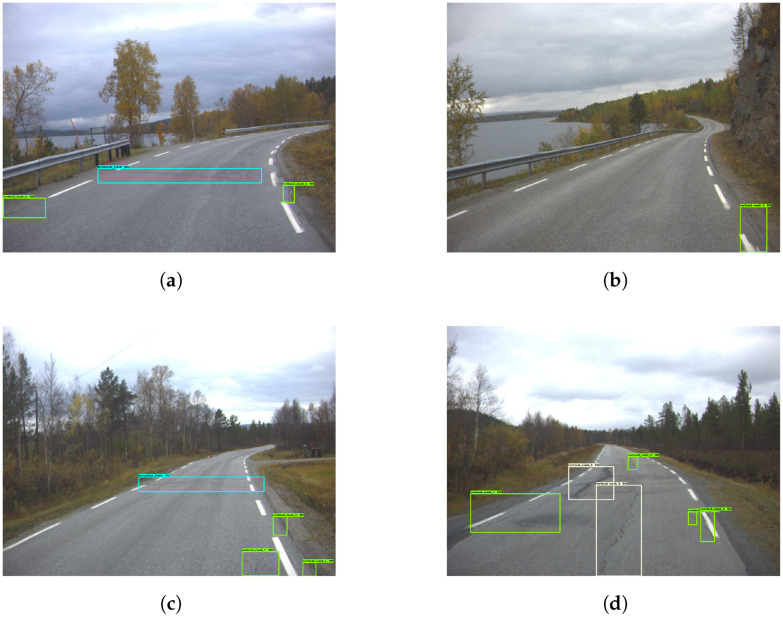
Good damage detection—Model 4. The green and yellow boxes represent the vertical cracks while cyan colored boxes are the horizontal cracks.

**Figure 16:**
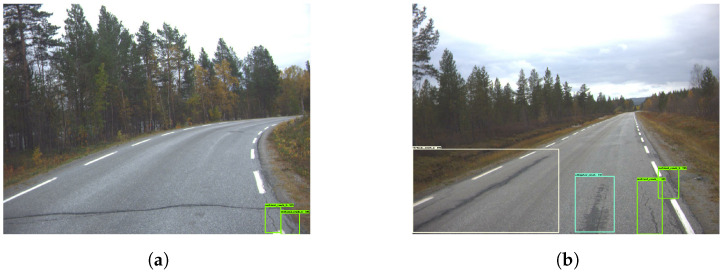
Missed damage detection—Model 4. The green and yellow boxes represent the vertical cracks while cyan colored boxes are the horizontal cracks.

**Figure 17:**
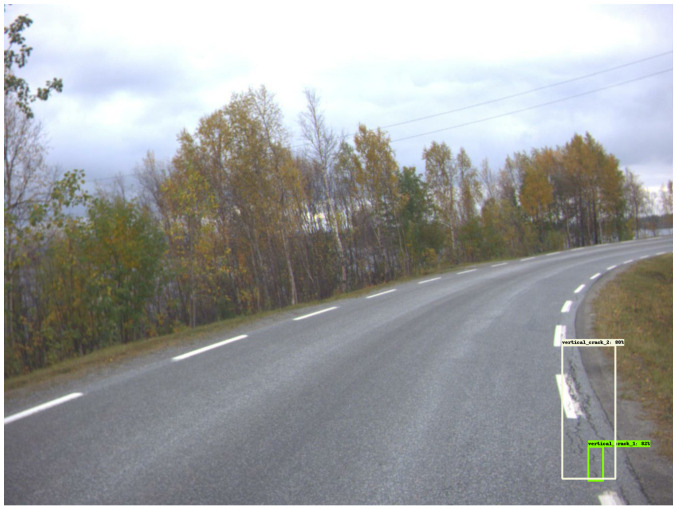
Mixed boxes—Model 4. The green box represents the vertical cracks while cyan colored box is the horizontal cracks.

The model produced several good evaluation images, as seen in Figure 15. Figure 15a,c,d indicates good capability of correctly detecting different classes while also being precise with labels. However, in Figure 16a, an example is shown where the model missed a distinct horizontal crack. In Figure 16b, the model wrongly detected a wet part of the road as an ‘alligator crack’. Figure 17 provides an example of how the model can sometimes predict cracks within cracks.

#### 6.1.5. Model 5—SSD MobileNet V1 COCO

The SSD MobileNet V1 network pre-trained on the MS-COCO dataset was fine-tuned on Veidekke. All images were resized to 400×400 and augmented with random horizontal flips.

In Table 9, it can be seen that the model predicted vertical cracks correctly 68 times. It missed 92 times and wrongly classified 4 times. Alligator cracks were correctly predicted five times and wrongly predicted once. The precision, recall and F1-score of Model 5 are presented in Table 10.

The training lasted 4 h and 41 min, resulting in a mAP of 40%. The calculated precision and recall values of the fine-tuned model with a 50% IoU and confidence threshold, are shown in Table 11.

#### 6.1.6. Model 6—SSD Inception V2 COCO

The SSD Inception V2 model was pre-trained on the MS-COCO dataset and fine-tuned on Veidekke. All images were resized to a 400 × 400 resolution and augmented with random horizontal flips.

After 3.5 h of training and 20,000 timesteps, it achieved 20% mAP. The calculated precision and recall with 50% confidence and IoU threshold are depicted in Table 11. The corresponding confusion matrices, at the same thresholds, are listed in Table 12.

#### 6.1.7. Model 7—SSD MobileNet V1 COCO

Model 7 was pre-trained on the MS-COCO dataset and fine-tuned on P18. The model achieved 5% mAP. As seen in Table 13, most damages were not detected by the model. Table 14 shows a low recall score that indicates that the model rarely detects damages. Conversely, when the damages are detected, the model has high classifying precision.

### 6.2. VGG16

**Figure 18:**
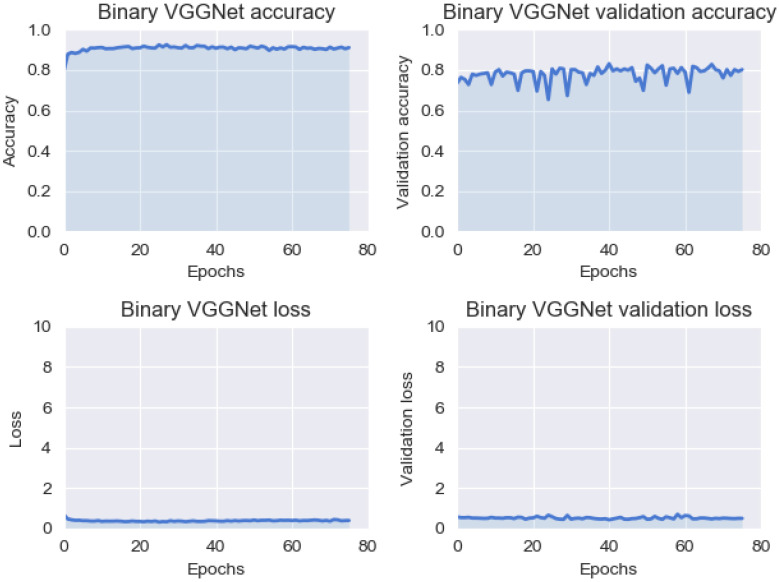
VGG16 binary training statistics. The statistics for the model remain close to static throughout the training phase.

The following results are achieved using the Veidekke dataset split into two classes for binary classification. Results from the pre-trained VG166 network can be seen in Figure 18 and in Table 15 and Table 16. The model miss-classified 105 out of 261 images in Table 15.

In Table 16, the ‘Damage’ precision is low, while ‘no damage’ is much higher. This is also present in the F1-score, which represents the combination of precision and recall. The F1-score is given by Equation (3).

### 6.3. Autoencoder

#### Convolutional Autoencoder

During the testing of the first model, the center layer, also known as the latent space representation, had a resolution of 16 × 16.

**Figure 19:**
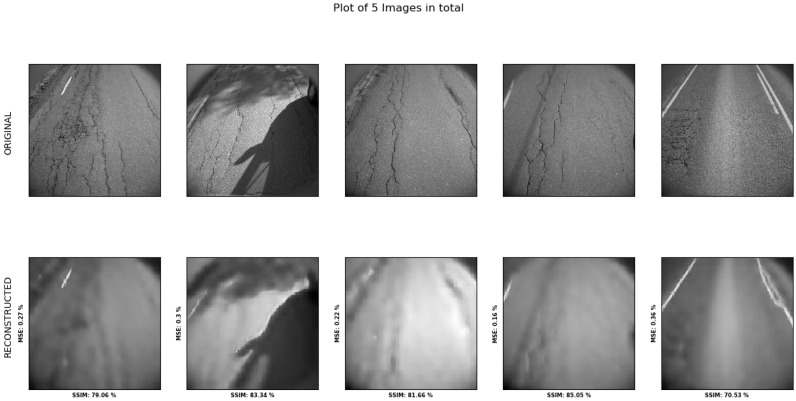
Model performance of reconstruction visualized, with the bottom road showing the reconstructed images and the top row showing the original image. This involves tests of no-, low-, and medium-damage classes.

The reconstruction is blurry, as seen in Figure 19.

Table 17 shows the results sampled from five images of each class. It indicates that there is little to no difference in the reconstruction error between classes. The * indicates the images with more than half the surfaces covered in shadows.

Table 18 shows the SSIM averaged over 150 samples for each of the four classes. Differences between classes are marginal. Training the model for an additional 10 epochs shows very small changes in the overall SSIM, as witnessed in Table 19.

Using the same five sampled images from each class as before, the results shown in Table 20 indicate little to no change.

**Figure 20:**
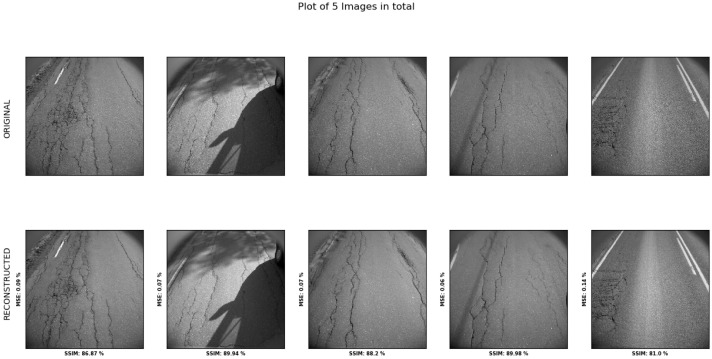
Model performance with adjusted hyperparameters visualized, with the bottom road showing the reconstructed images and the top row showing the original image. These are for tests of no-, low-, and medium-damage classes.

Changing the hyperparameters of the model to increase the latent space representation resolutions shows a clearer reconstruction of the image in Figure 20.

The reconstruction scores seen from the image are shown in Table 21. The averaged SSIM value in Table 22 indicates small differences between classes, with ‘high damage’ scoring slightly higher than the three others.

Training the model for another 10 epochs showed a slight increase in reconstruction accuracy for all categories in Table 23.

## 7. Discussion

Through transfer learning, object detection has shown promising results regarding automatic damage detection. The fine-tuned models in this work were able to detect damages. However, the overall accuracy was lacking, even though the models might have been interpreted as accurate when inspecting validation images.

Two object detection approaches were tested, faster R-CNN and SSD. Both were pre-trained on different datasets, fine-tuned on different datasets, and used different meta-architectures. Faster R-CNN is generally considered slow and precise, while SSD provides faster and less accurate predictions.

Through inspection of predicted images, the faster R-CNN model predictions are good. The SSD network, model 5, had the highest mean average precision, but it overlooked several damage types entirely.

Some of the inconsistencies in all models were from the dataset. Both datasets had small amounts of data, and some classes were underrepresented in the datasets. In the P18 dataset, all models struggled to determine the difference between the two vertical cracks. Furthermore, most models fine-tuned on the Vegdekke dataset failed to learn the underrepresented classes properly.

In this work, seven object detection models were fine-tuned to detect road surface damage. Four models consisted of a faster R-CNN network, while the other three used SSD networks. All models were able to recognize damages to different degrees. A comparison between them can be seen in Table 24. In this section, we discuss several of the key elements within object detection models. The most prominent are as follows:

- The dataset used for pre-training.
- The dataset used for fine-tuning.
- The meta-architecture of the models.

Section 7.1 discusses how the meta-architecture influences results. In Section 7.2, we discuss the choice of pre-training datasets and how it affects the results. The datasets and how the tanning data impacts this work are briefly discussed in Section 7.3. Finally, Section 7.4 draws all topics together to discuss the model’s performance compared to the goal of this work.

### 7.1. Meta-Architectures

Throughout this work, two meta architectures were used for object detection. SSD and faster R-CNN are two well-known architectures commonly used in the research community. We discuss different meta-architectures and how they affect the results in the following subsections.

Faster R-CNN is considered a precise architecture with low computational times. New approaches, such as SSD, further improve the detection speed by reducing the computational cost. SSD outperforms faster R-CNN in most situations. Smaller objects are the only exception where R-CNN can compete, even beating SSD in some cases.

The models in Table 24 are compared regarding the generated mAP scores. Admittedly, mAP will not accurately measure a model’s performance. As described earlier, mAP estimates a model’s precision. The limitation of this metric is that it only considers how well-predicted boxes fit with the ground-truth boxes. The mAP score ignores damages that are missed by the predictions. In addition, the imbalanced datasets can affect the mAP if the model only learns some of the classes. The impacts of the datasets are discussed in Section 7.3.

Seven models were trained in this work, three of which used SSD, while the remaining four used faster R-CNN. In order to properly compare the two architectures, the models should be compared when fine-tuned on the same dataset. As shown in Table 24, the highest mAP score at 40% was reached by Model 5 using SSD architecture. Likewise, the highest-scoring faster R-CNN model was Model 1, achieving a mAP of 30%. Both models were fine-tuned on Veidekke and pre-trained on MS-COCO. It should be noted that they did not use the same feature extractor.

In order to properly examine the models, the confusion matrices and evaluation images must be thoroughly inspected. When comparing Table 2 and Table 9, it is evident that Model 5 detected fewer cracks than Model 1 while having a higher precision. It is hard to determine why one seems more reserved than the other. It could be due to the architectural differences between the two models. In addition, it could be due to differences in the feature extractor. Ultimately, both models detect cracks and give decent representations of road conditions.

Given that mAP can be affected by imbalanced datasets, a closer look at the precision and recall for each class gives insight into the models. Table 1 and Table 10 show that both models properly learned how to predict vertical cracks.

Model 1 predicted 79% of the actual vertical cracks (recall), while 67% of the predicted vertical cracks were correct (precision). Converted to percent, Table 2 shows that 50% of alligator cracks and 100% of potholes were predicted as vertical cracks. If classes were ignored, Model 1 attained 76% recall and 70% precision. In comparison, Model 5 achieved 41% recall and 81% precision. If classes were ignored, the Model 5 recall increased to 45%, and precision increased to 86%.

When inspecting the two models, it becomes apparent that there is an optimization issue. One way of indicating which model performs better is to calculate the F1-score. This is a mathematical compromise between precision and recall. Due to the low number of damage in some classes, it is sensible to look at the accuracy score for each class separately. Table 1 shows that Model 1 achieved a 72% F1-score. Likewise, Table 10 shows Model 5 attained a 54% F1-score. Despite having a lower mAP, Model 1 achieved an overall better vertical crack detection performance than Model 5.

Due to using different feature extractors, it is difficult to measure how much the architecture affects these results. It is not unreasonable to assume that faster R-CNN’s heavier computing algorithms give it some advantages in the detection of smaller cracks. To see if this statement has any merit, the models trained on P18 must be inspected. Model 2 and Model 7 will be used to compare these two meta-architectures. Model 2 is identical to Model 1, except it is trained on P18 instead of Veidekke. Likewise, Model 7 is identical to Model 5 but trained on P18.

In the P18 dataset, images were taken close to parallel to the road surface. It captured both the road and the surrounding environment. This caused each image to contain less road surface. Thus, cracks appear smaller than in Veidekke. Typically, this would favor a faster R-CNN compared to the SSD architecture. Model 2 (faster R-CNN) achieved 20% mAP, a 10% reduction compared to the same network trained on Veidekke. Similarly, Model 7 (SSD) decreased by 35%, reaching a mAP of 5%. As stated earlier, mAP does not fully represent a model’s performance. Further inspection of the confusion matrices is required to understand how the two models perform.

The low recall scores in Table 14 indicate that Model 7 (SSD) struggled with detecting actual damages. Table 13 also shows that the vertical crack was only detected 10% of the time. Model 7 rarely predicted any damage; even when it did, it achieved a rather low accuracy. As a result, the F1-score of the vertical crack was only 17%.

Model 2 performed much better at detecting damages. Vertical 1, Vertical 2, and horizontal cracks achieved F1-scores of 57%, 52%, and 67%, respectively. Both models experienced performance hits when changing datasets. The dataset-specific changes are discussed in Section 7.3. It is interesting to see that Model 7, using the SSD architecture, had the highest drop in precision. SSD can struggle with smaller objects, which seems to be aligned with the decline.

As previously mentioned, comparing meta-architectures with different feature extractors is challenging. Even so, comparing the four models, the meta-architecture effects became apparent. SSD struggled when the size of the damages decreased. Moreover, faster R-CNN detected the most damage in both datasets. It was more sensitive, which caused images with considerable damage to be cluttered, leading to a lower precision. This was observable in evaluation images. The images containing the most damaged parts of the road were specifically handpicked to train and test the model. In a real-world application, inspection images often contained no or low amounts of visible damage. This could help make cluttering a negligible factor, thus increasing precision. When Model 1 and 2 start cluttering, the road might already be critically damaged. However, these models must undergo further testing in order to conclude whether the more sensitive models would increase their accuracy in a real-world application.

### 7.2. Pre-Trained Dataset

The pre-trained dataset is one of the main components of transfer learning. By training on a dataset, the outer layers learn to recognize features. When a model is fine-tuned, it may already know how to extract some features. The similarity between the datasets determines how much of the learned features are translatable. This determines how long a model has to be fine-tuned to learn the features of the new dataset. This sub-section discusses the relative impacts of the pre-trained dataset.

There are three different pre-trained datasets used in this work. In order to properly compare the effects of datasets used for pre-training, other variables must be taken into account. Model 2 and Model 4 are two R-CNN networks where the only difference is the pre-trained dataset, as seen in Table 24. This provides a sound basis for comparison.

First off, in Table 24, it can be seen that Model 2 pre-trained on MS-COCO achieved a 20% mAP. Model 3, pre-trained on the Oidv4 dataset, achieved a 14% mAP. This indicates that the difference in the score was caused by using different datasets in pre-training. By inspecting the two confusion matrices in Table 4 and Table 8, it is evident that Model 4 has a lower recall than Model 2. This suggests that Model 4 struggles with recognizing features when compared to Model 2.

In transfer learning, the model should eventually reach the same precision no matter the initialized weights, given that the dataset is big enough. If the dataset is small, the weights provided from pre-trained models will impact the performance of a fine-tuned model. The datasets used in this work are considered small. In order to examine the effects this might have on precision, further inspection is required.

Oidv4 contains approximately 9 million images, with 14.6 million annotated labels [42]. MS-COCO contains 300,000 images with 1.5 million object instances [10]. Admittedly, determining which transferred features help the models in this work is challenging. However, through logical reasoning, one could assume that by having similar features, images containing roads could be better suited. Since it is time-consuming to scour through all the millions of images, another assumption must be considered. Images of cars, motorcycles, buses, and trucks would typically include roads. Statistics for these categories are included in a graph provided by MS-COCO [10]. Oidv4 only provides a category called “vehicles”, which includes objects such as airplanes. However, those statistics did shed some light on the differences between the datasets.

Approximately 145,000 vehicle-related images were found in the MS-COCO dataset. This is estimated in their work [10]. Oidv4 is estimated to have a little under 100,000 images of vehicle “boxes” in their dataset, read from figures on their website [42]. This is a rough estimation. There are several images in both datasets that contain roads without vehicles. It becomes evident that MS-COCO contains about 50% more road vehicles than Oidv4. This can have caused the pre-trained model to learn more road-related features from MS-COCO. It is important to point out that each image of a road can contain a varying amount of vehicles. Regardless, the estimated occurrences of vehicles in the two datasets indicate, to some extent, how well-represented roads are.

Model 3 was trained with a different feature extractor compared to Models 2 and 4. This makes it hard to measure the effects of using the KITTI dataset. KITTI is commonly used in autonomous vehicle projects and consists only of images containing roads. However, the 2D object detection dataset is rather small, at only 14,999 images with 80,256 annotated objects [41]. Given the different feature extractors, no conclusion can be drawn on whether KITTI is better or worse than the other two datasets. When comparing the number of road objects in all three datasets, MS-COCO appears to have the most. It could indicate that MS-COCO is the better option for transfer learning regarding road damage detection. The data on the topic are inconclusive and are speculated. Even so, some of the differences in precision between Model 2 and Model 4 must be accounted for by the pre-trained dataset used.

### 7.3. Dataset

Datasets used during fine-tuning enable models to learn new classes quickly. Two datasets were created in this work. In this section, three elements are discussed. First, the annotation of datasets; second, how P18 compares to Veidekke; and third, how annotations and the two datasets impacted each model’s performance.

P18 contained 2483 images with a total of 6303 damages. Veidekke contained 626 images with 2113 damages. Even in the context of transfer learning, both datasets are small. It might be challenging for models to learn the important features (as discussed in Section 7.2). Veidekke was part of an experimental test setup and had limited data. Moreover, P18 is used in NPRA’s yearly road inspections, and they gathered several million images of roads. However, NPRA was amid the GDPR adjustments and could only provide a limited amount of images.

Both datasets were annotated using LabelImg. Veidekke was the first dataset to be annotated. Four classes were defined (as seen in Table 25). By following NPRA’s guidelines of damage classification, each of the damage classes labeled should have three degrees of severity. Given the small amount of data, there was no classification of severity in Veidekke. Five classes were defined when annotating P18, introducing severity 1 and 2 to the vertical crack class (as seen in Table 26).

The number of damage occurrences and consistency in annotations affect how a fine-tuned model performs. Since three non-professionals labeled both datasets, there is likely to be a degree of inconsistency. For instance, the distinction between severity 1 and 2 was loosely defined and highly subjective. This can make it challenging for fine-tuned models to separate between the degrees of severity within each class correctly. In the confusion matrix of Model 2 (Table 4), it can be seen that the model rarely mistakes ‘vertical crack 1’ for ‘vertical crack 2’. On the contrary, ‘vertical crack 2’ is often mistaken for ‘vertical crack 1’. As mentioned, the model might suffer from inconsistent labeling. Conversely, due to the similar nature of both vertical cracks 1 and 2, it is reasonable to expect some wrong predictions between the two damages. Increasing the amount of data may help the model learn to separate the severity degrees better.

The Veidekke dataset does not contain enough instances of potholes, alligator cracks, and horizontal cracks for the model to learn correctly. When inspecting Model 1’s confusion matrix (Table 2), it can be seen that neither potholes nor horizontal cracks are predicted. Moreover, alligator cracks are inconsistent, with one wrong, three correct, and three miss-classified predictions. Comparing Model 1 with Model 5 shows that neither models were able to properly learn potholes and horizontal cracks. Model 5 achieved a 62% F1-score for alligator cracks. Due to only six alligator cracks in the evaluation set, it does not provide a good basis to conclude how a model would perform. The model may have learned that alligator cracks consist of several small vertical cracks. Given the above, it is reasonable to propose that Veidekke either needs fewer classes or more data to improve precision.

The volume of each class in P18 (Table 26) is skewed toward ‘vertical crack 1’. However, the overall number of damages is increased compared to Veidekke. The confusion matrices of the P18 models (Table 4, Table 6 and Table 8) show all five classes being learned. The models still struggle to distinguish between classes. For instance, ‘alligator crack’ and ‘vertical crack 2’ are often entangled with ‘vertical crack 1’. This might be due to the skewed balance of the training data. Moreover, the lower image resolution can make it difficult to distinguish between damages.

Naturally, any road dataset will contain a larger amount of vertical cracks than other damages. Potholes are rare because they are critical to road safety and will often be fixed immediately. In future datasets, it may be worth gathering more data to increase the amount of rare road distresses.

Veidekke has a higher amount of pixels defining road surface than P18. The images in Veidekke have a resolution of 2046×2046, while the images in P18 have 2703×1018 pixels. Combined with the fact that roads cover larger portions of images in Veidekke, they provide more details than P18. This might account for the higher accuracy achieved in Veidekke models. A single degree of severity in vertical cracks can also give Veidekke an advantage.

Cha et al. [43] found that CNN performance improvements stagnated after 10,000 training images. While their research was based on a CNN, it may be reasonable to assume that an object detection model would need similar amounts of data. This suggests that both Veidekke and P18 need increased amounts of data. Maeda et al. [44] gathered their dataset containing 163,664 road images, 9053 of which were annotated with 15,435 bounding boxes. This led to high precision on several types of damages using an SSD MobileNet model. As discussed in this section, both datasets have great potential for improvement by adding more data.

It is revealed in this section that Veidekke holds two key advantages over P18;

- Higher image resolution.
- Road covering the entire image.

Even so, the Veidekke dataset was under-represented. In order to properly test the strengths and limitations of both datasets, further research should be conducted with additional data.

### 7.4. Performance

Determining how well the models perform can be difficult. Section 7.2 and Section 7.3 discussed how certain aspects affected the model performances. How the models performed compared to the goals of this work have yet to be discussed. Before work on the work started, a few goals were created:

- Models should be accessible through a server.
- Models should be able to detect several types of road distress.

The model could be used as an integrated service on any online platform to achieve these goals. Calculating a road surface condition index would also be possible through the model.

All models can be served through TensorFlow Serving, as described in Section 4. The docker environment remained stable throughout testing and could potentially be used in production. The second goal was only partially successful. None of the models performed well enough to be integrated into a production solution. Several issues must be solved before they are ready for use.

Given that rare damages are severely under-represented in Veidekke, the mAP scores are not representative. Instead, the precision of the classes should be considered. Model 1 achieved the highest precision for vertical cracks with 50% average precision and a 73% F1-score. Given the above, it is likely that Veidekke has the most significant potential for road damage detection.

## 8. Conclusions

The NPRA collects roughly five million images of road surfaces in Norway during their yearly surveys. Inspecting the images has primarily been conducted manually. In this work, we developed a PdM-based road inspection framework in which the DL models were applied to analyze these images to provide a concrete overview of road conditions. The detection and classification of road distress damages can be achieved using object detection methods. All models were poor at differentiating between classes and degrees of severity. This problem can be reduced or rectified by providing a more substantial dataset. Several key factors were found to affect the performance of the models; meta-architecture, dataset, and transfer learning. Faster R-CNN has the highest sensitivity and was, therefore, able to identify smaller damages. The models trained on Veidekke achieved the best precision, despite having fewer training samples. Using a specific pre-trained model improved detection capabilities. The practical application of the model was simplified by using a website as an interface. It allowed multiple images to be uploaded and rendered the accumulation of damage in a heatmap.

Many architectures have not been tested as yet, such as the MASK R-CNN approach. This combines object detection and semantic segmentation in a single output. Semantic segmentation can help define the area of damage. This area can then be used to calculate a percentage of the road that contains the damage. Combining this with the previous classification of object detection, it may be possible to develop a ‘fleshed out’ metric to determine the level of decay in a road. The NPRA already has aggregated data for 1 km stretches with the following metrics:

- Unevenness measured in the international roughness index mm/m.
- Rutting measured in mm.
- Cross-fall measured in percentage.

Combining this with results from a deep neural network can provide valuable insight into road conditions.

Since data were collected over multiple years, it may be possible to create a model that looks at the same stretches of roads over a longer period of time. The goal of this approach should be to identify the rate of decay. Given such a rate, it is feasible to calculate an estimated time for the remaining useful life of the road surface.

## Figures and Tables

**Table 1:** Evaluation results of Model 1.

Damage	Precision@0.5IOU	Recall@0.5IOU	F1-Score@0.5IOU
Vertical crack	67%	79%	73%
Horizontal crack	NaN	0%	NaN
Alligator crack	75%	50%	60%
Pothole	NaN	0%	NaN

**Table 2:** Confusion matrix of Model 1.

Actual	Predicted				
	Vertical	Horizontal	Alligator	Pothole	None
Vertical	130	0	0	0	34
Horizontal	0	0	0	0	9
Alligator	3	0	3	0	0
Pothole	2	0	0	0	0
None	58	0	1	0	0

**Table 3:** Table for precision and recall Model 2.

Damage	Precision@0.5IOU	Recall@0.5IOU	F1-Score@0.5IOU
Vertical crack 1	66%	51%	57%
Vertical crack 2	55%	50%	52%
Horizontal crack	66%	70%	68%
Alligator crack	18%	33%	23%
Pothole	43%	26%	32%

**Table 4:** Confusion matrix of Model 2.

Actual	Predicted					
	Vertical 1	Horizontal	Alligator	Pothole	Vertical 2	None
Vertical 1	598	11	56	4	35	480
Horizontal	4	89	1	0	2	32
Alligator	12	0	19	1	1	24
Pothole	3	1	0	6	0	13
Vertical 2	46	7	2	0	86	31
None	244	27	27	3	33	0

**Table 5:** Precision and recall of Model 3.

Damage	Precision@0.5IOU	Recall@0.5IOU	F1-Score@0.5IOU
Vertical crack 1	72%	42%	53%
Horizontal crack	77%	48%	59%
Alligator crack	23%	28%	25%
Pothole	80%	17%	28%
Vertical crack 2	63%	47%	54%

**Table 6:** Confusion matrix of Model 3.

Actual	Predicted					
	Vertical 1	Horizontal	Alligator	Pothole	Vertical 2	None
Vertical 1	497	8	43	0	28	608
Horizontal	8	62	1	0	3	54
Alligator	10	0	16	0	1	30
Pothole	2	0	0	4	0	17
Vertical 2	44	1	2	1	81	43
None	130	10	9	0	16	0

**Table 7:** Precision and recall of Model 4.

Damage Type	Precision@0.5IOU	Recall@0.5IOU	F1-Score@0.5IOU
Vertical crack 1	72%	38%	50%
Horizontal crack	79%	32%	46%
Alligator crack	19%	23%	21%
Pothole	100%	13%	23%
Vertical crack 2	56%	51%	53%

**Table 8:** Confusion matrix of Model 4.

Actual	Predicted					
	Vertical 1	Horizontal	Alligator	Pothole	Vertical 2	None
Vertical 1	449	6	33	0	39	657
Horizontal	2	41	0	0	3	82
Alligator	9	0	13	0	3	32
Pothole	1	1	1	3	1	16
Vertical 2	38	1	4	0	87	42
None	122	3	16	0	22	0

**Table 9:** Confusion matrix of Model 5.

Actual	Predicted				
	Vertical	Horizontal	Alligator	Pothole	None
Vertical	68	0	4	0	92
Horizontal	2	1	0	0	6
Alligator	1	0	5	0	0
Pothole	1	0	0	0	1
None	12	0	1	0	0

**Table 10:** Precision and recall Model 5.

Damage Type	Precision@0.5IOU	Recall@0.5IOU	F1-Score@0.5IOU
Vertical crack 1	81%	41%	54%
Horizontal crack	100%	11%	20%
Alligator crack	50%	83%	62%
Pothole	NaN	0%	NaN

**Table 11:** Precision and recall Model 6.

Damage Type	Precision@0.5IOU	Recall@0.5IOU	F1-Score@0.5IOU
Vertical crack 1	92%	29%	44%
Horizontal crack	NaN	0%	NaN
Alligator crack	100%	17%	29%
Pothole	NaN	0	NaN

**Table 12:** Confusion matrix of Model 6.

Actual	Predicted				
	Vertical	Horizontal	Alligator	Pothole	None
Vertical	47	0	0	0	117
Horizontal	0	0	0	0	9
Alligator	3	0	1	0	2
Pothole	0	0	0	0	2
None	1	0	0	0	0

**Table 13:** Confusion matrix of Model 7.

Actual	Predicted					
	Vertical 1	Horizontal	Alligator	Pothole	Vertical 2	None
Vertical 1	120	2	0	0	2	1060
Horizontal	8	3	0	0	0	117
Alligator	7	0	1	0	0	49
Pothole	0	0	0	4	0	19
Vertical 2	26	3	0	0	21	122
None	25	0	0	1	6	0

**Table 14:** Precision and recall for Model 7.

Damage Type	Precision@0.5IOU	Recall@0.5IOU	F1-Score@0.5IOU
Vertical crack 1	65%	10%	17%
Horizontal crack	38%	2%	4%
Alligator crack	100%	2%	4%
Pothole	80%	17%	28%
Vertical crack 2	72%	12%	21%

**Table 15:** Confusion matrix for VGG16 binary classification.

	Predicted	
	Damage	No damage
Damage	15	60
No damage	45	141

**Table 16:** Classification report from testing on 261 images.

Condition	Precision	Recall	F1-Score	Support
Damage	0.25	0.20	0.22	75
No damage	0.70	0.76	0.73	186
Macro average	0.48	0.48	0.48	261
Weighted average	0.57	0.60	0.58	261

**Table 17:** Sampled results for five images per class.

Samples	No Damage		Low Damage		Medium Damage		High Damage	
	MSE	SSIM	MSE	SSIM	MSE	SSIM	MSE	SSIM
1	0.22	77.07	0.22	77.41	0.24	77.18	0.27	79.06
2	0.21	78.64	0.23	80.11	0.22	78.66	0.22	81.66
3	0.14	86.17	0.15	84.51	0.18	82.13	0.16	85.05
4	0.28	74.43	0.15	81.57	0.37	69.24	0.36	70.53
5*	0.17	86.76	0.11	89.66	0.16	85.25	0.3	83.34

**Table 18:** Average mean over 150 samples.

	No Damage	Low Damage	Medium Damage	High Damage
**SSIM**	0.76897	0.78254	0.78887	0.81260

**Table 19:** SSIM averaged over 150 samples for a model training 20 epochs. Δ indicates the change in the results given by Δ = results 20 epochs − results 10 epochs.

	No Damage	Low Damage	Medium Damage	High Damage
**SSIM**	0.76745	0.78532	0.78851	0.81507
Δ	−0.00152	+0.00278	−0.00036	+0.00247

**Table 20:** Sampling of images with a slight difference in results for five images per class.

Samples	No Damage		Low Damage		Medium Damage		High Damage	
	MSE	SSIM	MSE	SSIM	MSE	SSIM	MSE	SSIM
1	0.21	77.19	0.22	77.4	0.24	77.17	0.27	79.06
2	0.15	82.19	0.22	80.12	0.22	78.75	0.22	81.65
3	0.14	86.19	0.14	84.61	0.18	82.13	0.16	85.04
4	0.23	74.54	0.15	81.56	0.36	69.28	0.34	70.56
5*	0.16	86.79	0.1	89.72	0.16	85.31	0.3	83.42

**Table 21:** Hyper reconstruction of images from each class.

Samples	No Damage		Low Damage		Medium Damage		High Damage	
	MSE	SSIM	MSE	SSIM	MSE	SSIM	MSE	SSIM
1	0.1	84.5	0.09	85.57	0.09	85.71	0.09	86.87
2	0.07	88.19	0.07	89.56	0.08	86.64	0.07	88.2
3	0.05	90.72	0.06	89.56	0.07	88.26	0.06	89.98
4	0.11	82.71	0.07	87.1	0.15	79.98	0.14	81.0
5*	0.04	91.91	0.03	93.37	0.05	90.63	0.07	89.94

**Table 22:** SSIM averaged over 150 samples for a modified model (training 10 epochs).

	No Damage	Low Damage	Medium Damage	High Damage
**SSIM**	0.85113	0.84675	0.86361	0.88097

**Table 23:** Final reconstruction of images from each class with a slight difference of results.

Samples	No Damage		Low Damage		Medium Damage		High Damage	
	MSE	SSIM	MSE	SSIM	MSE	SSIM	MSE	SSIM
1	0.08	87.23	0.08	88.35	0.07	88.44	0.07	89.31
2	0.05	90.29	0.06	89.92	0.06	89.25	0.06	90.32
3	0.04	92.35	0.05	91.43	0.06	90.34	0.05	91.75
4	0.09	85.85	89.29	0.06	0.13	83.72	0.11	84.58
5*	0.03	93.44	0.03	94.47	0.04	92.26	0.06	91.67

**Table 24:** Comparison of the fine-tuned object detection models.

Name	Architecture	Feature Extractor	Pre-Trained Dataset	Dataset	mAP
Model 1	Faster R-CNN	Inception ResNet V2 Astrous	MS-COCO	Vegdekke	30%
Model 2	Faster R-CNN	Inception ResNet V2 Astrous	MS-COCO	P18	20%
Model 3	Faster R-CNN	ResNet 101	KITTI	P18	15%
Model 4	Faster R-CNN	Inception ResNet V2 Astrous	Oidv4	P18	14%
Model 5	SSD	MobileNet V1	MS-COCO	Vegdekke	40%
Model 6	SSD	Inception V2	MS-COCO	Vegdekke	20%
Model 7	SSD	MobileNet V1	MS-COCO	P18	5%

**Table 25:** Damage occurrences in the Veidekke dataset.

Crack Types	Training	Evaluation
Vertical crack	1765	164
Horizontal crack	66	9
Alligator crack	85	6
Pothole	16	2

**Table 26:** Damage occurrences in the P18 dataset.

Crack Types	Training	Evaluation
Vertical crack 1	3530	1184
Vertical crack 2	392	172
Horizontal crack	503	128
Alligator crack	233	57
Pothole	81	23

## Data Availability

The data presented in this study are available upon request from the corresponding author.
